# Axillary shoot proliferation and plant regeneration in *Euryodendron excelsum* H. T. Chang, a critically endangered species endemic to China

**DOI:** 10.1038/s41598-020-71360-9

**Published:** 2020-09-01

**Authors:** Shuangyan Chen, Yuping Xiong, Teng Wu, Kunlin Wu, Jaime A. Teixeira da Silva, Youhua Xiong, Songjun Zeng, Guohua Ma

**Affiliations:** 1 Guangdong Provincial Key Laboratory of Applied Botany, South China Botanical Garden, The Chinese Academy of Sciences, Guangzhou, 510650 China; 2 University of Chinese Academy of Sciences, Beijing, 100039 China; 3 Zhongkai University of Agriculture and Engineering, Guangzhou, 510225 Guangdong China; 4 Retired, Miki-cho, Ikenobe 3011-2, Kagawa-ken 761-0799 Japan

**Keywords:** Biotechnology, Cell biology

## Abstract

*Euryodendron excelsum* H. T. Chang is a single-type, rare and endangered woody plant unique to China. In this study, young stems were used as explants and cultured on Woody Plant Medium (WPM) supplemented with 5.0 μM 6-benzyladenine (BA), were subcultured for more than 15 times over a total of more than 3 years and finally an efficient axillary shoot proliferation and plantlet regeneration system was established in which one shoot could proliferate an average of 5.1 axillary shoots every 2 months on the medium supplemented with 5.0 μM BA and 0.5 μM α-naphthaleneacetic acid (NAA). Shoots rooted at a moderate frequencies (50.1%) on agarized WPM supplemented with 0.5 μM NAA but 100% of shoots rooted in agar-free vermiculite-based WPM after culture for 2 months. Plantlets, when transplanted to peat soil: vermiculite (1:1), showed the highest 95.1% survival within 1 month.

## Introduction

*Euryodendron excelsum* H. T. Chang is a perennial tree and a monotypic genus endemic to China in the subfamily Ternstroemoideae in Theaceae^1,2^, Now it has been removed from Theaceae to form Pentaphylacaceae together with Pentaphylax^3,4^. *E. excelsum* is distributed in the south of China, and is native to Yangchun city, Guangdong Province and Pingnan County and Bama County of Guangxi Province^5,6^. In recent decades, population surveys of the species have found that *E. excelsum* is mainly distributed in rural areas with frequent human activities, unlike most other rare and endangered plants that are distributed in forest areas, so it is directly exposed to human activities^7,8^. Therefore, interference by human activity is the main cause of its endangerment^9^. In Pingnan County, *E. excelsum* was exterminated due to human interference and destruction of the living environment; In Yangchun city, only two *E. excelsum* ancient trees are alive^8^. *E. excelsum* has been listed as a National Protected Endangered Plant and is also classified as an extremely small species^10,11^. According to the definition of the International Union for Conservation of Nature (IUCN), *E. excelsum* is a critically endangered species^12,13^.

Natural renewal of *E. excelsum* populations is slow since young individuals are susceptible to harsh environmental factors, which, coupled with their own lack of survival competitiveness, make it difficult for them to replenish the population^14^. Therefore, it is imperative to protect *E. excelsum* seed resources. At present, the protection of *E. excelsum* depends mainly on efforts by local protection measures that promote the reproduction and renewal of natural populations. Relocation and reintroduction of endangered species, not only to protect and restore populations, provides scientific theoretical guidance and also offers protection to the biodiversity of China’s rare and endangered plant species^15,16^.

In natural communities, seed reproduction is the main method of reproduction^17,18^. A seed germination test found that *E. excelsum* seeds do not have a period of dormancy. After fruits were collected, seeds need to be sown immediately since there is a rapid loss of seed moisture that causes its germination rate (56%) to gradually decrease to 5%^17^. When cuttings (12 cm long with 6–7 nodes) of axillary shoots from 2-year-old *E. excelsum* were dipped in a solution of an auxin, or optimal rooting agent that contained 100 mg/L of indole-3-butyric acid (IBA), survival rate and rooting rate were only 20% and 16%, respectively^19^. When epicotyl and stem sections were used as explants for in vitro culture, few axillary shoot buds were induced, the highest shoot proliferation coefficient (SPC; number of new shoots/number of old shoots) was only 1.83 after subculture every 2 months for 2 years. However, no rooting or transplanting was reported^20^. In this study, *E. excelsum* axillary shoots were successively subcultured over more than 3 years to achieve an efficient shoot proliferation and rooting for the first time for this rare and critically endangered endemic Chinese tree species, laying down a solid foundation for its protection and sensible utilization.

## Results

### Effect of subculture period on shoot proliferation

**Figure 1:**
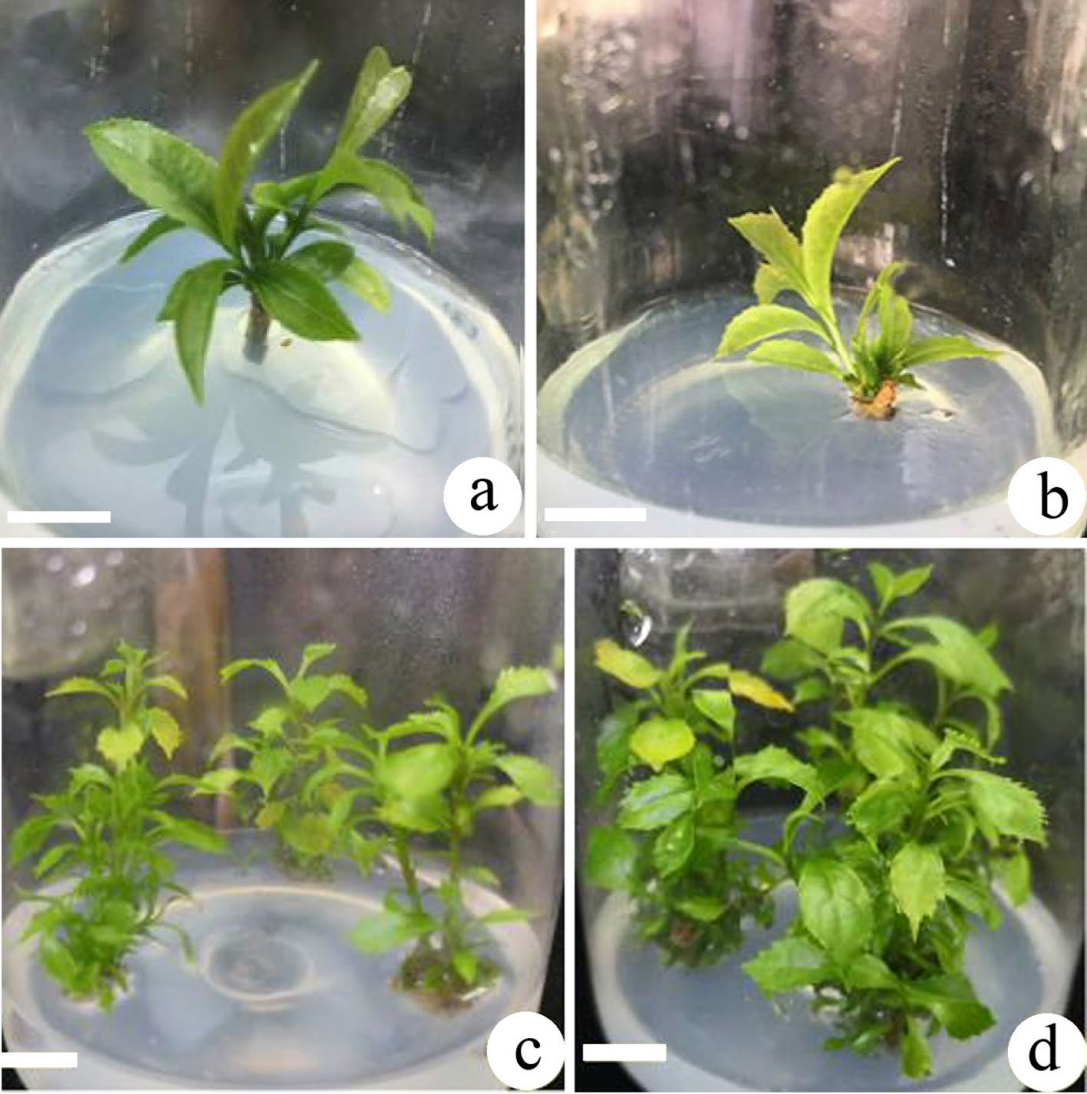
Axillary shoot propagation on the WPM. (**a**) The stem was cultured on the PGR-free medium for 2 months and the nodes developed new axillary shoots; (**b**) Multiple shoots begin proliferated on the WPM supplemented with 5.0 μM BA for 2 months in early stage; (**c**,**d**) Multiple shoots were propagated on the WPM supplemented with 5.0 μM BA and 0.5 μM NAA for 1 and 2 months at later stage, respectively. Bars = 1.0 cm.

In the early stage, very few axillary shoots were proliferated (1–2 shoots) (Fig. 1a). The subcultures were prolonged on WPM supplemented with 5.0 μM BA for more than 3 years and they generally developed multiple shoot clumps (Fig. 1b). The SPC could reach to 4.7 within 2 months.

As the multiple shoot clumps were transferred to different media, the SPC showed different. 1–10 μM Kinetin (KIN) induced SPC was usually between 2 and 3 (Table 1), while 1–10 μM BA-induced SPC generally reached 4–5 indicating that BA induction effect of SPC is stronger than KIN. The higher of KIN concentration, the higher the induced SPC. Similarly, the higher the BA concentration, the higher the induced SPC to 2.8 in 1 month (Fig. 1c) and 5.1 in 2 months (Fig. 1d). As BA concentration reached to the level of 5–10 μM, SPC is basically flat. When BA and NAA are combined in the WPM, the SPC seemed improve to some extent, but overall, there is no significant differences.

**Table 1:** Effects of PGRs on shoot proliferation coefficient (SPC) of *Euryodendron excelsum.*

PGRs (μM)	Shoot proliferation coefficient
KIN 1.0	2.1 ± 0.2 d
KIN 5.0	2.4 ± 0.2 d
KIN 10.0	2.8 ± 0.2 c
BA 1.0	3.5 ± 0.3 b
BA 5.0	4.7 ± 0.4 a
BA 10.0	4.6 ± 0.4 a
BA 5.0 + NAA 0.5	4.9 ± 0.4 a
BA 5.0 + NAA 1.0	5.1 ± 0.4 b
BA 10.0 + NAA 1.0	5.0 ± 0.4 a

Shoot proliferation and subculture on WPM supplemented with 5.0 μM BA and 0.5 μM NAA once every 2 months. Each treatment has 30 shoots. Different letters within a column indicate significant differences according to Duncan’s multiple range test (*P* < 0.05).

### Effect of PGRs and medium base on root induction

**Figure 2:**
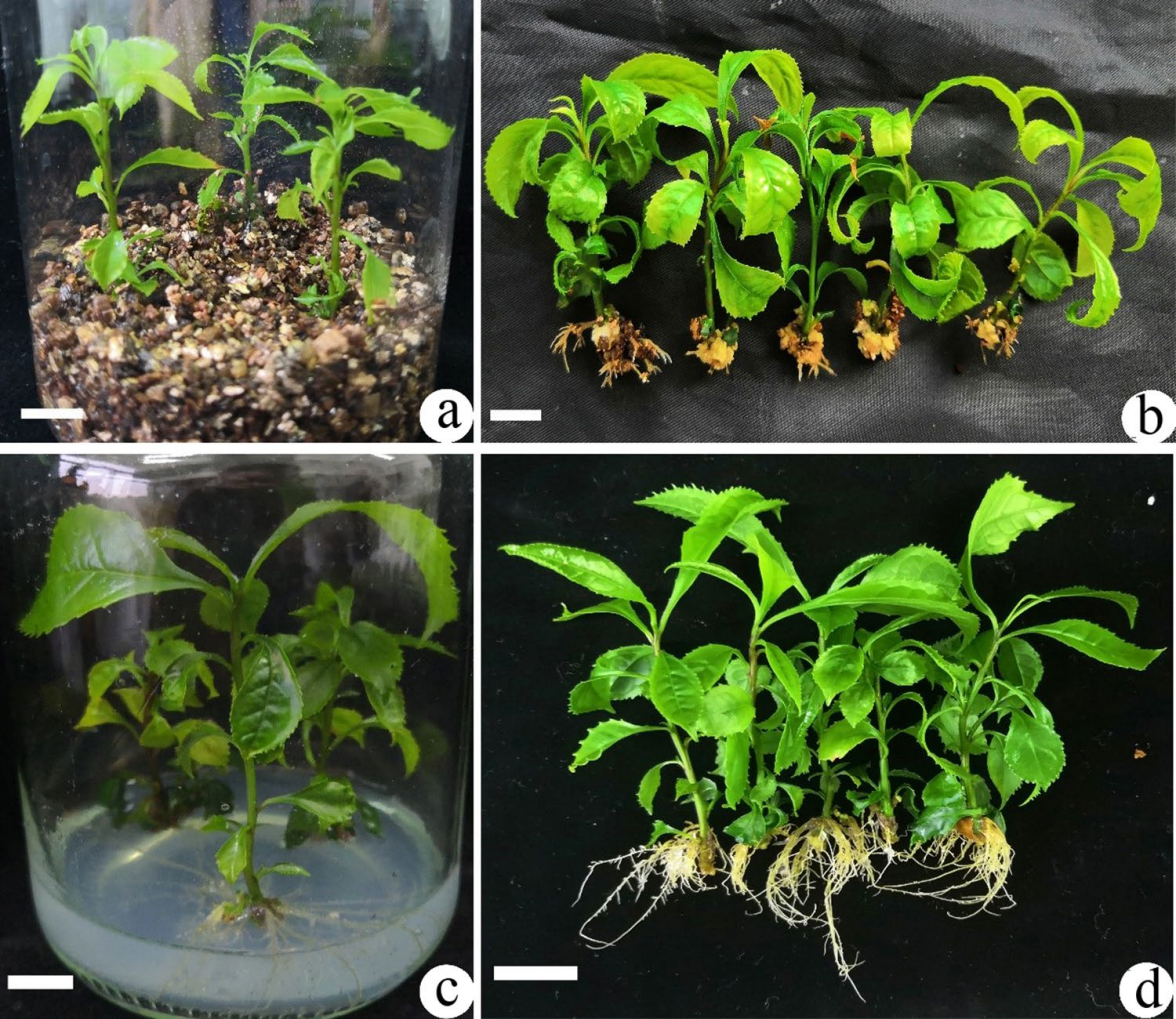
Rooting of *Euryodendron excelsum* on WPM supplemented with 10.0 μM IBA and 0.5 μM NAA. (**a**,**b**) Rooting in vermiculite-based WPM for 2 months in which some callus developed at the base of shoots. (**c**,**d**) Rooting on agar-based WPM for 3 months showing a more developed root system free of callus, relative to A and B. Bars = 1.0 cm.

In vermiculite-based WPM (Fig. 2a), when 0.5–10 μM IBA was used alone, the highest rooting percentage was 48.5%, when 0.5–10 μM NAA alone used, the highest rooting percentage increased to 50.1%, and when IBA was combined with NAA, rooting percentage was 100% (Table 3) and some callus formed at the shoot bases (Fig. 2b). However, in agar-based WPM (Fig. 2c), when IBA was used alone, rooting percentage was less than 8.7%, when NAA was used alone, it increased to 12.7%, while the IBA and NAA combination resulted in 21.5% rooting (Table 2, Fig. 2d).

**Table 2:** Effects of PGRs in WPM on rooting of *Euryodendron excelsum* after 2 months.

PGRs (μM)	Rooting percentage (%)	
	Vermiculite	Agar
IBA 0.5	11.3 ± 1.2 d	0 ± 0 e
IBA 2.5	28.2 ± 2.1 c	5.3 ± 1.3 d
IBA 10.0	48.5 ± 2.0 b	8.7 ± 1.7 cd
NAA 0.5	50.1 ± 9.8 b	4.3 ± 1.5 d
NAA 2.5	33.3 ± 9.5 c	6.3 ± 1.2 d
NAA 10.0	23.7 ± 4.8 c	12.7 ± 2.0 c
IBA 5.0 + NAA 0.5	100 ± 0 a	4.7 ± 0.7 d
IBA 7.5 + NAA 0.5	100 ± 0 a	5.0 ± 1.0 d
IBA 10.0 + NAA 0.5	100 ± 0 a	48.5 ± 1.8 a
IBA 0.5 + NAA 2.5	100 ± 0 a	6.7 ± 1.2 d
IBA 0.5 + NAA 5.0	100 ± 0 a	21.5 ± 1.2 b
IBA 0.5 + NAA 7.5	100 ± 0 a	9.0 ± 1.2 cd

Each treatment has 30 shoots. Different letters within a column indicate significant differences according to Duncan’s multiple range test (*P* < 0.05).

### Acclimatization and transplantation

**Figure 3:**
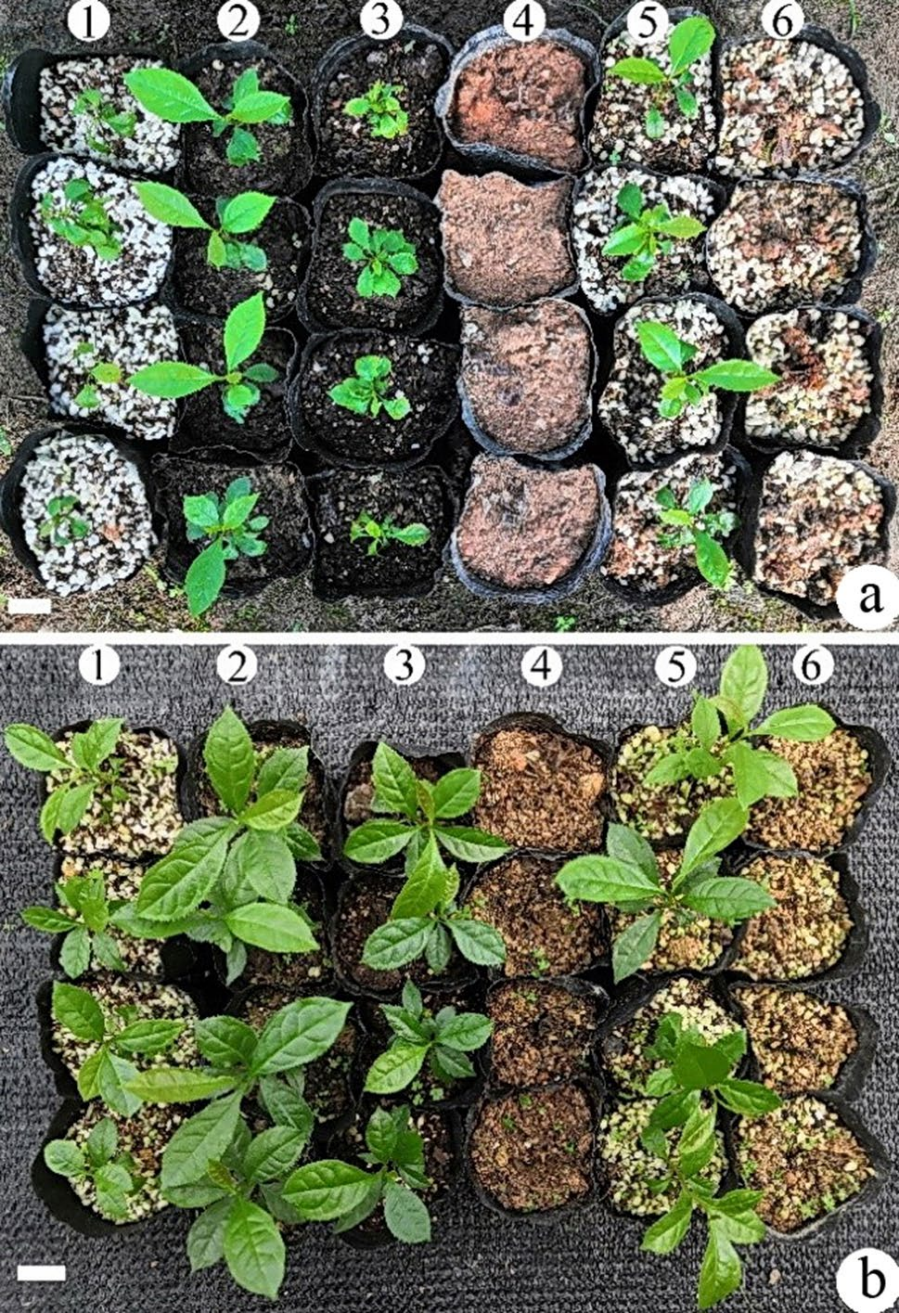
Acclimatization and transplanting of *Euryodendron excelsum.* Bars = 2.0 cm. (**a**) Rooted plantlets were transplanted in different substrates for a month. (1) Vermiculite: pearl rock (1:1); (2) peatsoil: sand (1:1); (3) peat soil: vermiculite (1:1); (4) peatsoil: yellow mud (1:3); (5) peatsoil: yellow mud: vermiculite (1:2:1); (6) yellow mud: pearl rock: peatsoil (1:1:1). (**b**) Rooted plantlets were transplanted to different substrates for 3 months. (1) vermiculite: pearl rock (1:1); (2) peatsoil: sand (1:1); (3) peat soil: vermiculite (1:1); (4) peatsoil: yellow mud (1:3); (5) peatsoil: yellow mud: vermiculite (1:2:1); (6) yellow mud: pearl rock: peatsoil (1:1:1).

30 days after transplanting *E. excelsum* plantlets with roots, plantlets showed highest survival (> 95%) in peat soil: vermiculite (1:1). In substrates of peat soil: yellow mud: vermiculite (1:2:1) and peat soil: sand (1:1), survival percentage exceeded 82%, and in vermiculite: pearl rock (1:1), it was 60%. In substrates of peat soil: yellow mud (1:3) and yellow mud: pearl rock: peat soil (1:2:1), no transplanted plants survived (Table 3, Fig. 3a). 90 days after transplanting *E. excelsum* plantlets with roots, highest survival (> 84%) was in peat soil: vermiculite, > 74% in peat soil: yellow mud: vermiculite (1:2:1) and peat soil: sand (1:1), and 55% in vermiculite: pearl rock (1:1). In peat soil: yellow mud (1:3) and yellow mud: pearl rock: peat soil (1:2:1) substrates, no transplanted plants survived (Table 3, Fig. 3b).

**Table 3:** Transplanting of *Euryodendron excelsum* in different substrates for 1/3 months.

Plantlets transplanting substrates (volumetric ratios)	Survival percentage (%)	
	One month	Three months
Vermiculite: pearl rock (1:1)	60.0 ± 2.9 c	55.0 ± 2.9 c
Peatsoil: sand (1:1)	82.4 ± 1.7 b	78.3 ± 1.2 b
Peatsoil: vermiculite (1:1)	95.1 ± 1.2 a	84.7 ± 2.0 a
Peatsoil: yellow mud (1:3)	0 ± 0 d	0 ± 0 d
Peatsoil: yellow mud: vermiculite (1:2:1)	82.1 ± 2.1 b	74.7 ± 2.0 b
Yellow mud: pearl rock: peatsoil (1:1:1)	0 ± 0 d	0 ± 0 d

Each treatment has 30 plantlets or shoots. Different letters within a column indicate significant differences according to Duncan’s multiple range test (*P* < 0.05).

## Discussion

In the early stage, shoot proliferation was usually much low. With the subculture times prolonged, more and more axillary shoots were induced from one shoot node. One shoot node could proliferate for 5.1 shoots every 2 month. This high SPC could never been extravagant wished 2 years before. This maybe that the level of cytokinins in the stem at an early stage is relatively low, so the number of induced axillary shoots is less, as the number of subcultures increased, endogenous cytokinin levels presumably increased, thereby causing to proliferate more axillary shoot buds. Through more than 3 years long term and successive tissue culture, we finally established an efficient shoot proliferation system (Table 1).

In the family Pentaphylacaceae, there was no report about tissue culture. In the former family, Theaceae, tea [*Camellia sinensis* (L.) O. Kuntze] has a very high economic value. Micropropagation have been reviewed, providing comprehensive accounts of the success and limitations of biotechnological tools applied to tea and its wild relatives^19,20^. The embryogenic callus of *Camellia nitidissima* Chi. could differentiate into somatic embryos, nodular embryogenic structures and adventitious shoots depending on the PGR used in WPM. BA was best for adventitious buds^21^. The effect of cytokinins and GA_3_ as well as different sucrose concentrations (5, 10, 20, and 30 g/L) on axillary shoot multiplication of *Camellia japonica* L. was investigated. High quality shoots and highest multiplication coefficient (3.4 shoots/basal explant; 2.4 shoots/apical explant) were obtained on WPM medium supplemented with BA, TDZ and GA_3_^22^. Through 3 years subculture and test optimal PGRs in axillary shoots and rooting conditions. We established an efficient shoot proliferation, rooting and transplanting system. It may be the most successful establishing an efficient multiple shoot proliferation system in the family Theaceae. This will laid a better foundation for the future proliferation, biotechnology and preservation in *E. excelsum.*

Vermiculite is sometimes used in tissue culture for rooting and transplanting^23^. In this experiment, vermiculite is much more effective than agar on rooting medium (Table 2). On the vermiculite-based WPM rooting media, rooting percentage could reach 100%. However, on agar-based media, maximum rooting was < 48.5%. Adding vermiculite to transplanted substrates improves rooting percentage and plantlet survival due to increased aeration. In contrast, almost all plantlets in substrates with yellow mud could not survive, so *E. excelsum* needs a well-aerated substrate for plantlets transplantation and recovery.

## Materials and methods

### Selection and culture of explants

Young stems of *E. excelsum* were collected from several 18-year-old trees growing on a mountainside in the Magnolia Garden of the South China Botanical Garden, Guangzhou. The sample seedling trees were brought back from the habitat, Yangchun City and transplanted and appraised by our colleague Prof. Huagu Ye (first author in 6th reference)^6^. The *E. excelsum* seedlings had been approved by local forestry permission. The stems 7–8 cm long with 2–3 nodes were disinfected in 0.1% mercuric chloride (HgCl_2_) solution for 12 min then washed five times with sterile distilled water. Then the stems were cut into 2–3 cm long with one node were placed on an ultra-clean workbench and air-dried, and then inoculated onto agar (Solarbio, Beijing)-solidified plant growth regulator (PGR)-free half-strength Woody Plant Medium for new axillary shoot development^24^. In the early stage, every culture jars (12 cm high; 10 cm diameter) contained only one stem (Fig. 1a). After culture in light for a total of 2 months on this medium, the stems developed new axillary shoots and then transferred to new WPM supplemented with 5.0 μM 6-benzyladenine (BA) for multiple shoot proliferation (Fig. 1b). The WPM was supplemented with 20 g/L sucrose and 6.0 g/L agar, and medium pH was adjusted to 5.8–6.0 with 1.0 N HCl or 1.0 N NaOH. All the media was sterilized at 105 kPa and 121 °C for 20 min. Culture jars were placed in a 25 ± 1 °C culture room under a 12-h photoperiod with a photosynthetic photon flux density of 80 μM m^−2^ s^−1^ emitted by 40 W fluorescent lights (Philips, Tianjing, China). After that, the culture jar contained 3 multiple shoot clumps were subcultured onto the same WPM every 2 months. The shoots were continuously subcultured for more than 3 years with subculture number increasing to more than 18 times and generally developed multiple shoots. These multiple shoots could begin the following tests.

### Effect of PGRs on axillary shoot proliferation

The axillary shoots (buds) was cut into 2–3 shoots (buds) and then cultured on the new WPM media supplemented with different combinations of PGRs (Table 1). After culturing for 2 months, the shoot proliferation coefficient (SPC) was assessed as number of new shoots/number of old shoots.

### Effect of vermiculite in culture medium and auxins on root induction

Individual shoots 3–5 cm tall with 6–8 leaves were selected and cut from the base of axillary multiple shoot clusters and inoculated onto WPM containing agar or 11.0 g vermiculite. WPM was supplemented with different concentrations of indole-3-butyric acid (IBA) and α-naphthaleneacetic acid (NAA) for root induction (Table 2). Each treatment contained 30 axillary shoots (10 jars; 3 shoots/jar). After culture for 2 months, rooting percentage was assessed as: (number of shoots forming roots/total number of shoots) × 100%.

### Acclimatization and transplantation

Plants that rooted well in vermiculite-based culture (Table 2), transferred to several substrates (Table 3). All the vermiculite and perlite substrates were bought from Guangzhou Shunxin Company, China. Rooted plantlets were transplanted into black plastic bags (12 cm high; 10 cm diameter) which were filled to the top with substrates. Each bag contained only one plant. Bags were sprayed with tap water (~ 100 ml) every morning. One and three months after transplantation, survival percentage was calculated as: number of plantlets survival/total transplanting plantlets.

### Statistical analyses

Experimental data were statistically analyzed in SPSS17.0 software (IBM). Means were separated by analysis of variance and data in tables is represented by the mean ± standard error. Duncan’s multiple range test (DMRT) was used to assess significant differences between means (*P* < 0.05). Experiments were repeated in triplicate.

## Data Availability

All data generated or analyzed during this study are included in this published article.
